# Lifetime adverse pregnancy outcomes and associated factors among antenatal care booked women in Central Gondar zone and Gondar city administration, Northwest Ethiopia

**DOI:** 10.3389/fpubh.2022.966055

**Published:** 2022-08-10

**Authors:** Atalay Goshu Muluneh, Melaku Hunie Asratie, Tesfamichael Gebremariam, Aynalem Adu, Mihretu Molla Enyew, Endeshaw Admasu Cherkos, Senetsehuf Melkamu, Martha Berta, Worku Mamo, Dawit Kassahun, Nuhamin Tesfa Tsega, Azmeraw Ambachew Kebede, Desale Bihonegn Asmamaw, Getahun Molla Kassa, Muhabaw Shumye Mihret

**Affiliations:** ^1^Department of Epidemiology and Biostatistics, Institute of Public Health, College of Medicine and Health Sciences, University of Gondar, Gondar, Ethiopia; ^2^Department of Women's and Family Health, School of Midwifery, College of Medicine and Health Science, University of Gondar, Gondar, Ethiopia; ^3^Department of Surgical Nursing, School of Nursing, College of Medicine and Health Sciences, University of Gondar, Gondar, Ethiopia; ^4^Department of Clinical Midwifery, School of Midwifery, College of Medicine and Health Science, University of Gondar, Gondar, Ethiopia; ^5^North Gondar Zonal Health Department, Carter Center Zonal Project Coordinator, Gondar, Ethiopia; ^6^Department of Obstetrics and Gynecology, School of Medicine, College of Medicine and Health Sciences, University of Gondar, Gondar, Ethiopia; ^7^Department of Reproductive Health, Institute of Public Health, College of Medicine and Health Sciences, University of Gondar, Gondar, Ethiopia

**Keywords:** lifetime, adverse outcomes, pregnancy, factors, Ethiopia

## Abstract

**Background:**

In developing countries, adverse pregnancy outcomes are major public health issues. It is one of the leading causes of neonatal morbidity and mortality worldwide. Despite the fact that ending prenatal mortality and morbidity is one of the third Sustainable Development Goals (SDG), the burden of the problem continues to be a huge concern in developing countries, including Ethiopia. Hence, this study aimed to determine the prevalence and associated factors of lifetime adverse pregnancy outcomes among antenatal care (ANC) booked women in Northwest Ethiopia.

**Methods:**

An institutional-based cross-sectional study design was conducted in Northwest Ethiopia, between March 2021 and June 2021. A multi-stage stratified random sampling technique was employed to recruit participants. An interviewer-administered and checklist questionnaire were used to collect the data. The data were entered into Epi-data version 4.6 software and exported to Stata version 16 for analysis. The binary logistic regression model was fitted to identify an association between associated factors and the outcome variable. Variables with a *p*-value of < 0.05 in the multivariable logistic regression model were declared as statistically significant.

**Results:**

In this study, the lifetime prevalence of adverse pregnancy outcome among study participants was 14.53% (95%CI: 11.61, 18.04). Road access to the health facilities (AOR = 2.62; 95% CI: 1.14, 6.02) and husband-supported pregnancy (AOR = 2.63; 95 CI: 1.46, 4.72) were significantly associated with adverse pregnancy outcomes.

**Conclusions:**

More than one in 10 reproductive age women had adverse pregnancy outcome throughout their life. Road access to health facilities and husband-supported pregnancy were statistically significant factors for adverse events in pregnancy. Therefore, it is better to give more attention to expanding infrastructure like road accessibility and increasing husband-supported pregnancy to reduce adverse pregnancy outcomes.

## Background

Pregnancy is a delightful natural gift when it is completed without any problems or grief. However, if the result is unsatisfactory, it could be an unpleasant experience for the mother, even for the whole family (1). Stillbirth, premature delivery, low birth weight, and birth defects are some of the adverse pregnancy outcomes (2). These adverse pregnancy effects will not be limited to death but also cause long-term physical and neurological impairments for the survived babies (3).

Despite substantial efforts made to decrease adverse pregnancy outcomes, a large number of pregnancies remain complicated with disastrous results. Regarding this, in the year 2014, the World Health Assembly authorized a target aimed at lowering the worldwide stillbirth rate to 12 per 1,000 births by the year 2030 in all countries (4). However, the stillbirth rate is alarmingly rising. Globally, ≈2 million pregnancies resulted in stillbirth (i.e., one stillbirth every 16 s) (5), 98% of these occurring in resource-limited countries (6). Of these, about 0.88 million stillbirths have occurred in Sub-Saharan Africa (SSA) and 24.6 stillbirth rate per 1,000 total births in Ethiopia (5).

Along with stillbirth, low birth weight, congenital anomalies, and premature deliveries, these are public health issues that cause profound depression, loneliness, and economic impact on women and their families (7). Low birth weight complicates about 15–20% (2 million deliveries per year) around the world (8). As a result, the World Health Organization (WHO) established a strategy to reduce the global rate of low birth weight (LBW) by 30% by the year 2025 (9).

Most pregnancy adverse outcomes might have been avoided if women had received high-quality preconception, pregnancy, and delivery care (10, 11). Many adverse pregnancy outcomes are the result of poor quality care during pregnancy and childbirth, a lack of emphasis on prevention treatments, a failure to recognize the social consequences of adverse pregnancy outcomes, and a poor data recording system (5). Thus, to effectively manage adverse pregnancy outcomes, there is a need for contemporary data on the causes (12).

Multiple factors like older maternal age (13), experiencing delays in getting treatment (14), high-risk pregnancy (15, 16), lack of antenatal care visits (17), multiple pregnancies (18), and having chronic medical conditions (19) are some of the identified factors that can affect the adverse pregnancy outcome. Thus, adverse pregnancy outcomes can be prevented and treated with immunization, nutritional supplementation, and routine screening for specific diseases (20).

Ethiopia has implemented different strategies to decrease adverse pregnancy outcomes. Despite this, adverse pregnancy outcomes in Ethiopia remain significantly higher because predictors are poorly understood and prevention strategies are inadequate (21–23). Identifying the probable causes linked to poor pregnancy outcomes and developing a practical strategy would thus be beneficial in reversing the alarming trend. Therefore, this study aimed to assess the lifetime prevalence of adverse pregnancy outcomes and associated factors in northwest Ethiopia.

## Methods

### Study design, period and setting

An institutional-based cross-sectional study design was applied. The study was performed in central Gondar (Central Gondar zone and Gondar city administration), Northwest Ethiopia, from the period of March 2021 to June 2021. The Central Gondar zone is located 738 km northwest of Addis Ababa. The central Gondar includes the Central Gondar Zone with 15 districts and the Gondar City administration. According to the national reports conducted by the Central Statistical Agency of Ethiopia, Central Gondar has a total population of 2,711,329, of which 91,372 pregnancies were projected for 2020/21 by the zonal statistical agency. In the study area, maternal health services like antenatal care (ANC), skilled delivery, and postnatal care (PNC) services are freely available for women without any cost. All health centers and hospitals give maternal and neonatal health services.

### Source population and study population

All ANC booked pregnant women in the central Gondar zone and Gondar City were the source population. All ANC booked pregnant women in the selected health facilities were the study population. All women who had at least one normal live birth or adverse pregnancy outcome history before and were booked for ANC at the selected health facilities were included in our study. All women who had at least one normal live birth or adverse pregnancy outcome history before and were booked for ANC at the selected health facilities with mothers who died and critically ill were excluded.

### Sample size determination and sampling procedure

The sample size for the present study was calculated using a single population proportion formula by considering the following assumptions: 95% level of confidence, 18% proportion of adverse pregnancy outcome (19), and 5% margin of error.


(1) $$n=\frac{Z{\left(\alpha /2\right)}^{2}p(1-p)}{d^{2}}=n=\frac{{\left(1.96\right)}^{2^{*}}0.18\;(1-0.18)}{{\left(0.05\right)}^{2}}=227$$

Where, n = required sample size, α = level of significant, z = standard normal distribution curve value for 95% confidence level = 1.96, p = proportion of adverse pregnancy outcome, and d = margin of error. After considering a non-response rate of 10% and a design effect of 2, we obtained a total sample size of 500. A multistage stratified sampling technique was used to select the study participants. At the first stage, three districts ( Gondar zuria, West Dembia and Wogera) and one city administration were selected by using lottery methods, and from each district, 20% of the health facilities were selected. From the selected districts and Gondar city, five health facilities (160 participants), four health facilities (145 participants), two health facilities (75 participants), and three health facilities (88 participants) were selected from Gondar zuria district, Gondar city, West Dembia district, and Wogera district, respectively.

### Variables of the study

The dependent variable was life time adverse pregnancy outcome whereas the independent variables were age, marital status, education, residence, religion, occupation, and parity, number of living children, insurance membership, distance to the nearest health facility, pregnancy wontedness, pregnancy supported by the husband, the reason for the first ANC, and history of chronic illness.

### Operational definition

Adverse pregnancy outcome: was considered as “yes,” if women had at least one of the following before her current pregnancy: still birth, abortion, intrauterine growth restriction, congenital anomalies, gestational hypertension disorders, gestational diabetes, and preterm birth (2).

#### Preterm birth

If born before 37 completed weeks of gestation but after 28 weeks of gestation or low birth weight (24).

#### Congenital anomalies

It defined as any abnormality of physical structure found at birth or during the first few weeks of life; or any irreversible condition existing in a child before birth in which there is sufficient deviation in the usually number, size, shape, location of any part, organ, and cell to warrant its designation as abnormal (25).

#### Stillbirth

If the infant died in the womb or during the intrapartum period after 28 weeks of gestation (26).

#### Abortion

Fetus removed or expelled from the uterus before 28 weeks or weighing < 500 g (27).

### Data collection tools and quality control

The data collection tool was developed by reviewing the literature (13, 17–19, 28–30). A structured, interviewer-administered and checklist questionnaire were employed to collect the data through face-to-face interviews and observing charts. The questionnaire was developed in English first, then translated to Amharic (the local language), and re-translated back to the English language to check its consistency. The questionnaire contains socio-demographic characteristics, maternity health services, and reproductive-related characteristics of the participants. A total of 18-trained midwives collected the data under the supervision of six MSc holders. Data collectors and supervisors were oriented and trained for 1 day, focusing on how to select and interview the participants. The questionnaires were pretested on 24 study participants (5%) and modifications were made according to the results of the pretest.

### Data processing and analysis

Data were checked for completeness and entered into Epi-data version 4.6 Statistical software and transferred to the Stata version 16 for further cleaning and analysis. Descriptive statistics were described using frequencies, percentages, mean and standard deviation, which were further presented using tables, and texts. Normality tests such as kurtosis and skewness were employed to see the normal distribution of the variables and to identify which summary measures were appropriate to use.

A binary logistic regression model was used with a cut-off *P*-value <0.25 and <0.05 in the bi-and multi-variable analysis respectively. Adjusted odds ratio with 95% confidence intervals was computed to see the presence of an association between dependent and independent variables. The 95% CI was used to declare the statistical association. We tested the chi-square assumption and model goodness of fit was tested using the Hosmer Lemeshow test. Besides, the multi-collinearity assumption was tested using pseudo variance inflation factor (VIF), and standard error. Thus, parity was excluded from the final analysis because of the significant multi-collinearity effect.

## Results

### Socio-demographic characteristic of the participants

A total of 468 women with a 93.6% response rate were involved in this study, of which 198 (42.49%) were in the age group of 26–30, with a median age of 28 (IQR = 25, 31) years. The majority of the mothers 443 (94.66%) were married, and 279 (59.62%) mothers were rural residents. Regarding educational status, 287 (61.32%) had no formal education (Table 1).

**Table 1:** Socio-demographic characteristics of study participants in Northwest, Ethiopia, 2021 (*n* = 468).

**Variable**	**Category**	**Adverse pregnancy outcome**		**Frequency (%)**
		**Yes**	**No**	
Age	16–20	7	37	44(9.44)
	21–25	8	96	104(22.32)
	26–30	31	167	198(42.49)
	Above 30	22	98	120(25.75)
Residence	Urban	18	171	189(40.38)
	Rural	50	229	279(59.62)
Current marital status	Married	64	379	443(94.66)
	Not married	4	21	25(5.34)
Educational status	No formal education	50	237	287(61.32)
	Grade 1–8	11	83	94(20.09)
	Grade 9–12	3	53	56(11.97)
	College and above	4	27	31(6.62)

*Other: protestant and catholic*.

### Obstetric related characteristic of the study participants

Among the study participants, 200 (49.57%) mothers were multi-gravidas. Nearly half (49.79%) of mothers had community-based health insurance membership. Regarding husband-supported pregnancy, the majority of (80.56) pregnancies were husband-supported pregnancies (Table 2).

**Table 2:** Obstetrics related characteristics of study participants in Northwest, Ethiopia, 2021 (*n* = 468).

**Variables**	**Category**	**Adverse pregnancy outcome**		**Frequency n (%)**
		**Yes**	**No**	
Parity	1	19	159	178(38.03)
	2–4	32	200	232(49.57)
	4–9	17	41	58(12.39)
Community based health insurance membership	Yes	36	197	233(49.79)
	No	32	203	235(50.21)
Distance to a nearest health facility	30 min−1 h	19	115	134(29.32)
	<30 min	48	275	323(70.68)
Road access to health facilities	Year round road	27	254	281(60.04)
	Dry weather road	14	48	62(13.25)
	No road for vehicles at	27	98	125(26.71)
Pregnancy supported by husband	Yes	43	334	377(80.56)
	No	25	66	91(19.44)
	Being sick	30	86	116(25.78)
Time of 1st ANC booking	Within 16 weeks	17	116	133(30.23)
	Late (after16 weeks)	38	232	270(61.30)
	Unknown	9	28	37(8.41)
ANC visit for their recent birth	Yes	48	321	369(78.85)
	No	20	79	99(21.15)

### Proportion of adverse pregnancy outcome

The study found that the lifetime prevalence of adverse pregnancy outcome among study participants was 14.53% (95%CI: 11.61, 18.04). Out of which, the commonest adverse pregnancy outcomes was abortion 33 (7.05%) followed by stillbirth 15 (3.21%) (Table 3).

**Table 3:** Summary of lifetime adverse pregnancy outcome, in Northwest, Ethiopia, 2021 (*n* = 468).

**Categories of adverse pregnancy outcome**	**Frequency**	**Percent**
No adverse pregnancy outcome	400	85.47
Abortion	33	7.05
Still birth	15	3.21
Cs delivery and/or complications	8	1.71
Still birth and Gestation HTN, DM	4	0.85
Bleeding disorders of pregnancy	4	0.85
Others	4	0.85

### Factors associated with adverse pregnancy outcome

In the multivariable logistic regression analysis, road access to health facilities and husband-supported pregnancy had a statistically significant association with adverse pregnancy outcome.

The odds of adverse pregnancy outcome among women who had no road access to the health facilities was 2.62 times (AOR = 2.62; 95% CI: 1.14, 6.02) higher than those with road access to the health facilities. The odds of adverse pregnancy outcome among women with no husband-supported pregnancy were 2.62 times (AOR = 2.63; 95 CI: 1.46, 4.72) higher than those women with a husband-supported pregnancy (Table 4).

**Table 4:** Binary logistic regression analysis for factors associated with adverse pregnancy outcome, Northwest, Ethiopia, 2021(*n* = 468).

**Variable**	**Category**	**Adverse pregnancy outcome**		**COR (95%CI)**	**AOR (95%CI)**
		**Yes**	**No**		
Education of the respondent	No formal education	61	320	1.28 (0.43, 3.81)	
	Primary education	3	53	0.38 (0.08, 1.84)	
	Secondary education and above	4	27	1	
Marital status	Unmarried	4	21	1.13 (0.37, 3.39)	
	Married	64	379	1	
Religion	Orthodox	65	368	2.56 (0.59, 10.99)	
	Protestant	2	3	4.83 (0.33, 17.34)	
	Muslim	1	29	1	
Occupation of the respondents	Housewife	56	307	2.18 (0.65, 7.35)	
	Private employed	9	57	1.89 (0.48, 7.46)	
	Government employed	3	36		
Parity	1	19	159	1	1
	2–4	32	200	1.34(0.74, 2.45)	0.98(0.48, 4.72)
	4–9	17	41	3.47(1.66, 7.26)	1.93(0.75, 4.67)
Road access to health facilities	Year round road	27	254	1	1
	Dry weather road	14	48	2.74(1.34, 5.61)	2.62(1.14, 6.02)^*^
	No road for vehicles at	27	98	2.59(1.45, 4.64)	2.09(0.99, 4.41)
Residence	Urban	18	171	1	1
	Rural	50	229	2.07(1.17, 3.68)	0.98(0.46, 2.08)
Pregnancy supported by husband	Yes	43	334	1	1
	No	25	66	2.94(1.68, 5.15)	2.63(1.46, 4.72)^*^
Age	15–20	7	37	2.27(0.77, 6.71)	2.10(0.69, 6.40)
	21–25	8	96	1	1
	26–30	31	167	2.23(0.98, 5.04)	2.30(0.94,5.66)
	Above 30	22	98	2.69(1.14, 6.35)	2.02(0.75, 5.48)
ANC visit for their recent birth	Yes	48	321	1	1
	no	20	79	1.69(0.95, 3.01)	1.22(0.65, 2.27)

*Hosmer and Leme-show test P=0.29;*

* *statistically significant*.

## Discussion

This study was conducted to assess the lifetime prevalence of adverse pregnancy outcome and associated factors among ANC booked women in Northwest, Ethiopia. The overall lifetime prevalence of adverse pregnancy outcome in the current study was found to be 14.53% (95%CI: 11.61, 18.04). This is in line with a study conducted in southern Ethiopia (13.9%) (31), Tanzania (18%) (32). However, this finding is higher than a systematic review conducted in Ethiopia 5.3% (33). The possible explanations may be due to the current study being at a one zone in which total populations are different when we compare with the national and gap in the year of study and a lot of strategies and work has been implemented since then to improve maternal and child health service utilization (34).

On the other hand, this finding is also lower than the findings of studies conducted in Hossana town, Ethiopia (24.5%) (35), Wollo zone, Ethiopia (31.8%) (17), Ghana (19%) (36), and Uganda (37%) (37). The possible reasons for this discrepancy may be the difference in the study setting, time gap, and socio-demographic variations. For instance, the study done in Ghana was hospital-based, unlike the present study, which was also conducted at health centers. Commonly, health centers are located in remote areas where the inaccessibility of infrastructure makes it difficult to provide service to remote areas and is attributed to socio-economic variations and the quality of maternal health services. In addition, the variation of nutritional and cultural practices may contribute to the observed variations.

In this study, we found that women who had no husband-supported pregnancy were more likely to have an adverse pregnancy outcome than those who had a husband supported pregnancy. This is supported by studies conducted in the United States (28), and Nepal (38). The possible explanation might be that those who had no husband-supported pregnancy have been shown to have effects on women's behaviors prenatally (39). Those who have no husband-supported pregnancy may experience stress, decrease social support, are less likely to enter preconception and prenatal care, and husband unsupported pregnancy is usually an unwanted pregnancy. Which may increase the likelihood of negative pregnancy outcomes (40–42). Furthermore, evidence suggests that an unsupported pregnancy by a husband has no effect on maternal health care service access and utilization, such as ANC, institutional delivery, and postnatal care. It can affect pregnancy and childbirth through responding to health problems that can occur during pregnancy (preeclampsia, preterm birth, and infection), seeking medical help, paying for transport, and allocating household resources (42, 43).

The odds of an adverse pregnancy outcome among women who had no road access to the health facilities was 2.62 times higher than those who had road access to the health facilities. This could be because inaccessibility of roads restricts utilization of basic maternal health services like ANC, health facility delivery, and postnatal care, which leads to an increased adverse pregnancy outcome (44–46). Poor accessibility of road and transport facilities also affects hospital supplies, often resulting in a stock-out of essential medicines. Impediment of accessing timely referral care, which exacerbates the cost of accessing maternal health care services, and pregnant women conveyed by inappropriate transport, which exacerbates the already existing poor health. Moreover, the inaccessibility of transport is also directly responsible for adverse pregnancy outcomes (47, 48).

## Conclusion

More than one in 10 reproductive-age women had an adverse pregnancy outcome at some point in their life. Road access to health facilities and husband-supported pregnancy were statistically significant factors for adverse events in pregnancy. Therefore, it is better to give more attention to expanding infrastructure like road accessibility and increasing husband-supported pregnancy to reduce adverse pregnancy outcomes.

## Data availability statement

The original contributions presented in the study are included in the article/supplementary files, further inquiries can be directed to the corresponding author/s.

## Ethics statement

The studies involving human participants were reviewed and approved by University of Gondar. Written informed consent to participate in this study was provided by the participants' legal guardian/next of kin.

## Author contributions

AM: Conceived and designed the study, performed analysis and writing original draft and critically evaluated and made progressive suggestions throughout the study. GK, TG, EC, WM, NT, AA, MA, and MM: Participated in the design, developing methods and critically reviewed the manuscript. AM, GK, AK, DK, SM, MB, and DA: Participated in the study design, data analysis, interpretation and draft manuscript. AM, MM, DK, and DA: Participated in the data entry, analysis, interpretation, and critically revise the manuscript. All authors have read and approved the final manuscript.

## Conflict of interest

The authors declare that the research was conducted in the absence of any commercial or financial relationships that could be construed as a potential conflict of interest.

## Publisher's note

All claims expressed in this article are solely those of the authors and do not necessarily represent those of their affiliated organizations, or those of the publisher, the editors and the reviewers. Any product that may be evaluated in this article, or claim that may be made by its manufacturer, is not guaranteed or endorsed by the publisher.

## Abbreviations

AOR: Adjusted Odds Ratio
ANC: Antenatal Care
CI: Confidence Interval
COR: Crude Odds Ratio
COVID-19: Coronavirus disease
WHO: World Health Organization.
